# Clinical characteristics and prognostic factors of *Clostridium perfringens* infection complicated by massive intravascular hemolysis in patients with hematologic diseases: a retrospective case series study

**DOI:** 10.3389/fmed.2026.1726461

**Published:** 2026-03-04

**Authors:** Weixiang Lin, Juan Feng, Zhihong Fang

**Affiliations:** Key Laboratory of Xiamen for Diagnosis and Treatment of Hematologic Malignancy, Department of Hematology, The First Affiliated Hospital of Xiamen University, Xiamen Hematology Medical Quality Control Center, Xiamen, China

**Keywords:** altered mental status, *Clostridium perfringens*, hematologic malignancies, intravascular hemolysis, prognosis

## Abstract

Clostridium perfringens (CP), an anaerobic Gram-positive bacterium, is commonly associated with food poisoning and gas gangrene. In rare instances, it can cause fatal massive intravascular hemolysis (MIH), a condition associated with exceedingly high mortality that poses a serious clinical challenge. A retrospective analysis was performed using a fatal case of T-lymphoblastic lymphoma/leukemia complicated by CP-associated acute hemolysis treated at our center in November 2024 and case reports of CP-associated MIH in hematologic patients from January 1987 to September 2025. A total of 23 eligible cases from our institution and published literature were included. The cohort consisted of 60.8% (*n* = 14) male patients, with a mean age of 45.87 ± 17.94 years. All patients presented with fever, hematuria was observed in 69.5% of patients, shock in 78.2%, and altered mental status (AMS) in 60.8%. The overall mortality rate was 73.9%, with a median survival time of 13.5 (6, 24) hours among non-survivors. AMS was identified as an independent risk factor for mortality (OR = 14.03, 95% CI: 1.19–165.08, *p* = 0.036). The pathogenic cascade, conceptualized as a “double-hit” model, is triggered by the synergistic action of α-toxin and θ-toxin. Together, they induce fulminant intravascular hemolysis and a systemic inflammatory response, culminating in organ injury. Although no specific therapeutics are available, immediate empirical combination antibiotic therapy (such as penicillin with clindamycin) is paramount. Adjunctive measures, including intensive care support and toxin removal strategies, are essential components of care. This study emphasizes the severity of CP-MIH in hematologic patients and identifies AMS as a key prognostic marker, underscoring the need for early intervention and further research into rapid diagnostics and targeted treatments.

## 1 Introduction

The genus *Clostridium* encompasses diverse anaerobic, spore-forming pathogens that challenge patient safety in multiple ways. Not only does *Clostridioides difficile* cause a spectrum of gastrointestinal disease (1), and *Clostridium botulinum* induce a life-threatening neuroparalysis (2), but some species, notably *Clostridium perfringens* (CP), can invade systemically with devastating consequences. CP infection exemplifies this severe phenotype, capable of progressing to clostridial sepsis complicated by massive intravascular hemolysis (MIH)—a medical emergency with exceptionally high mortality (3). The primary virulence factors implicated are α-toxin and θ-toxin, which synergistically drive hemolysis and consequent organ injury. Clinically, CP-MIH often presents with fever, hematuria, shock, and altered mental status (AMS), progressing to death within hours (3).

Patients with hematologic diseases, particularly malignancies, are at high risk for CP infection. This susceptibility stems from treatment-induced bone marrow suppression which results in neutropenia as well as compromised immunity, and damage to the gastrointestinal mucosal barrier which permits translocation of colonizing bacteria into the systemic circulation (4). In combination, these alterations create a permissive environment for CP to establish rapidly progressive and frequently lethal septic episodes (5). Despite the recognized severity of CP infections, there remains a substantial gap in the literature concerning the specific clinical features and prognostic factors of CP-MIH within this vulnerable population. Existing studies have largely been limited to sporadic case reports, resulting in a lack of comprehensive evidence to guide clinical practice.

The present study seeks to address this knowledge gap through a retrospective analysis of patients with hematologic diseases who developed CP infection complicated by MIH. By integrating data from both local case and published reports, this study aims to characterize the clinical profile of CP-MIH and identify prognostic factors influencing outcomes in this high-risk population. It is anticipated that these findings will enhance the understanding of CP-MIH and inform evidence-based strategies for early clinical intervention.

## 2 Materials and methods

### 2.1 Study design

This retrospective case series study was performed in compliance with the Declaration of Helsinki and approved by the Ethics Committee of The First Affiliated Hospital of Xiamen University. We integrated cases from our institution with those identified through a systematic literature review. A total of 23 eligible cases spanning from January 1987 to September 2025 were included in the analysis.

### 2.2 Case identification

#### 2.2.1 Local case

In November 2024, a patient with T-lymphoblastic lymphoma/leukemia was admitted to our center. During a period of chemotherapy-induced bone marrow suppression, the patient developed high-grade fever, AMS, and hemolytic anemia, culminating in death within 6 h of symptom onset. Postmortem blood culture confirmed *C. perfringens* infection.

#### 2.2.2 Literature-derived cases

A systematic search was conducted in PubMed, Web of Science, China National Knowledge Infrastructure, Wanfang Data, and VIP database using the keywords “*Clostridium perfringens*” AND “hemolysis” and their Chinese equivalents. Cases were included if they met all of the following criteria: (i). confirmed hematologic diseases, (ii). evidence of *C. perfringens* infection (positive culture or PCR-based detection), (iii). documented intravascular hemolysis. Exclusion criteria were: (i). infection occurring in patients without hematologic disease, (ii). *C. perfringens* infection without hemolytic manifestations. After manual screening, 22 eligible cases were identified. Combined with the local case, a total of 23 patients were included. Key clinical characteristics are summarized in Table 1 (6–26).

**TABLE 1:** Summary of clinical data from included cases (*N* = 23).

Year reported	Sex	Age	Protopathy	Hemoglobin (g/L)	Leukocyte (×10^9^/L)	Primary infection site	Gas gangrene	Hematuria	AMS	Shock	The time of etiological diagnosis	Time from onset to death (hours)	Outcome
1987 (6)	M	58	DLBCL	39	0.27	NA	No	Yes	Yes	No	Before death	NA	Death
1992 (7)	F	54	AML	23	0.5	Skin and soft tissue	No	Yes	Yes	Yes	After death	11	Death
1994 (8)	M	19	ALL	37	0.3	NA	No	No	Yes	No	After death	9	Death
1996 (9)	F	73	CLL	NA	NA	NA	NA	No	Yes	Yes	After death	NA	Death
1997 (10)	F	55	HL	34	0.2	Respiratory system	No	Yes	Yes	No	After death	4	Death
2002 (11)	M	43	DLBCL	52	3.6	Gastrointestinal tract	Yes	Yes	No	Yes	After death	36	Death
2004 (12)	M	74	AML	80	1.4	Gastrointestinal tract	No	Yes	Yes	Yes	After death	20	Death
2005 (13)	M	50	B-ALL	35	NA	Gastrointestinal tract	No	Yes	No	Yes	After death	NA	Death
2007 (14)	M	58	AML	NA	0.4	NA	No	Yes	No	Yes	After death	16	Death
2008 (15)	F	31	Neutropenia	63	1.04	Gastrointestinal tract	Yes	Yes	Yes	Yes	After death	60	Death
2012 (16)	M	14	T-ALL	NA	0.5	Skin and soft tissue	Yes	No	Yes	Yes	After death	72	Death
2014 (17)	M	37	AML	71	0.2	Biliary tract	No	Yes	No	Yes	NA	–	Survival
2016 (18)	M	32	AML	52	0.1	NA	No	Yes	No	No	24 h	–	Survival
2017 (19)	M	17	B-ALL	28	0.6	Gastrointestinal tract	Yes	No	Yes	Yes	After death	6	Death
2018 (20)	M	54	Neutropenia	51	17.8	Gastrointestinal tract	No	Yes	No	Yes	10 h	–	Survival
2019 (21)	M	48	AML	NA	0.1	NA	No	No	Yes	Yes	Before death	18	Death
2021 (22)	M	21	AML	33	0.04	Gastrointestinal tract	No	Yes	No	Yes	12 h	–	Survival
2021 (22)	M	42	AML	44	0.27	Gastrointestinal tract	No	Yes	No	Yes	Before death	24	Death
2022 (23)	F	68	MM	30	NA	Gastrointestinal tract	No	No	Yes	Yes	Before death	4 h	Death
2023 (24)	F	62	AML	49	NA	Gastrointestinal tract	No	Yes	Yes	Yes	After death	6	Death
2024 (25)	F	53	B-ALL	35	0.11	NA	No	Yes	No	No	39 h	–	Survival
2025 (26)	F	62	MAL	45	0.06	Biliary tract	No	Yes	Yes	Yes	NA	–	Survival
This case	F	30	T-LBL/ALL	21	0.92	Gastrointestinal tract	No	No	Yes	Yes	After death	6	Death

AML, acute myeloid leukemia; ALL, acute lymphoblastic leukemia; B-ALL, B-cell acute lymphoblastic leukemia; CLL, chronic lymphocytic leukemia; DLBCL, diffuse large B-cell lymphoma; HL, Hodgkin lymphoma; MPAL, mixed phenotype acute leukemia; T-ALL, T-cell acute lymphoblastic leukemia; T-LBL/ALL, T-lymphoblastic lymphoma/leukemia; MM, multiple myeloma; AMS, altered mental status; F, female; M: male; NA, not available.

### 2.3 Statistical analysis

Data analysis was conducted with SPSS 30.0 software. Normally distributed continuous variables are presented as mean ± standard deviation and compared by independent *t*-test. Non-normally distributed data are summarized as median with interquartile range (Q1, Q3) and compared by Mann-Whitney U test. Categorical variables are expressed as number (frequency, %) and were compared using the χ^2^ test or Fisher’s exact test, as appropriate. Prognostic factors were firstly screened by univariate analysis, and significant variables were subsequently entered into a binary logistic regression model for multivariable analysis. The independent effect of each variable was quantified by calculating the odds ratio (OR) with its corresponding 95% confidence interval (CI). A two-sided *P*-value < 0.05 was considered statistically significant.

## 3 Results

### 3.1 Clinical characteristics

A total of 23 patients were included in the analysis, comprising 14 males (60.8%) and nine females (39.1%), with a mean age of 45.87 ± 17.94 years. Underlying hematologic diseases were distributed as follows: acute myeloid leukemia (9 cases, 39.1%), lymphoid malignancies (10 cases,43.48%, including 3 B-ALL, 2 DLBCL, 1 ALL, 1 T-ALL, 1 T-LBL/ALL, 1 CLL, and 1 HL), neutropenia (2 cases, 8.6%), mixed phenotype acute leukemia (1 case, 4.3%), Hodgkin lymphoma (1 case, 4.3%), chronic lymphocytic leukemia (1 case, 4.3%), and multiple myeloma (1 case, 4.3%). All patients presented with fever; other common clinical manifestations included shock (18 cases, 78.2%), hematuria (16 cases, 69.5%), and AMS (13 cases, 56.5%). Suspected infection sources were gastrointestinal (11 cases, 47.8%), hepatobiliary (2 cases, 8.6%), lower limb soft tissue (2 cases, 8.6%), and respiratory tract (1 case, 4.3%); however, 7 cases (30.4%) had no identifiable source. Detailed clinical characteristics are summarized in Table 2.

**TABLE 2:** Clinical features of *Clostridium perfringens* (CP) infection with massive intravascular hemolysis (MIH) in 23 patients with hematologic disorders.

Characteristics		Overall (*n* = 23)
Demographics		
	Male gender (%)	14 (60.8)
	Age, mean ± SD	45.87 ± 17.94
Comorbidities		
	Acute myeloid leukemia (%)	9 (39.1)
	Acute lymphoblastic leukemia (%)	1 (4.3)
	B-cell acute lymphoblastic leukemia (%)	3 (13.0)
	Chronic lymphocytic leukemia (%)	1 (4.3)
	Diffuse large B-cell lymphoma (%)	2 (8.6)
	Neutropenia (%)	2 (8.6)
	Hodgkin lymphoma (%)	1 (4.3)
	Mixed phenotype acute leukemia (%)	1 (4.3)
	T-cell acute lymphoblastic leukemia (%)	1 (4.3)
	T-lymphoblastic lymphoma/leukemia (%)	1 (4.3)
	Multiple myeloma (%)	1 (4.3)
Clinical presentation		
	Gas gangrene (%)	4 (17.3)
	Hematuria (%)	16 (69.5)
	AMS (%)	14 (60.8)
	Shock (%)	18 (78.2)
Hemoglobin (g/L, mean ± SD)	–	43.26 ± 15.74
Leukocyte [×10^9^/L, median (Q_1_, Q_3_)]	–	0.285 (0.105, 0.760)
Source of infection		
	Gastrointestinal tract (%)	11 (47.8)
	Biliary tract (%)	2 (8.6)
	Skin and soft tissue (%)	2 (8.6)
	Respiratory system (%)	1 (4.3)
	Not available (%)	7 (30.4)
Hours to death [hours, median (Q_1_, Q_3_)]	–	13.5 (6, 24)
Mortality (%)	–	17 (73.9)

### 3.2 Survival outcomes and prognostic factors

Among the 23 patients, 6 cases (26.1%) survived, and 17 cases (73.9%) died. The median survival time for the fatal cases was 13.5 (6, 24) hours. Microbiological confirmation of CP infection was obtained postmortem in 76.4% (13/17) of fatal cases, while only 23.5% (4/17) were diagnosed antemortem. All etiological diagnoses were obtained exclusively from blood specimens. Univariate logistic regression analysis was performed to evaluate the association between clinical variables and mortality risk, including gender, age, white blood cell count, hemoglobin level, AMS, shock, and hematuria. The results indicated that AMS and WBC count were significantly statistically significant between non-survivors and survivors (Table 3). A significantly higher proportion of patients exhibited AMS in the deceased group compared to the survival group (76.5% vs. 16.7%; *P* = 0.010). Additionally, a potential upward trend in WBC count was observed, though with overlapping interquartile ranges (0.45 [0.25–0.95] vs. 0.11 [0.05–4.60] × 10^9^/L; *P* = 0.029). No other variables showed statistically significant associations with outcome (*P* > 0.05).

**TABLE 3:** Univariate comparison of clinical characteristics between the deceased group and the survival group.

Variable	Death	Survival	*t/*χ *2*	*P*-value
Gender, *n* (%)			0.12	0.735
Male	10 (58.82)	4 (66.67)		
Female	7 (41.18)	2 (33.33)		
Age (years)	46.82 ± 19.03	43.17 ± 15.64	0.42	0.678
Hemoglobin (g/L)	41.63 ± 14.42	47.83 ± 13.83	−0.13	0.368
WBC (× 10^9^/L)	0.45 (0.25, 0.95)	0.11 (0.05, 4.60)	−2.18	0.029*
Hematuria, *n* (%)			3.55	0.059
No	7 (41.18)	0 (0)		
Yes	10 (58.82)	6 (100)		
AMS, *n* (%)			6.66	0.010*
No	4 (23.53)	5 (83.33)		
Yes	13 (76.47)	1 (16.67)		
Shock, *n* (%)			0.64	0.423
No	3 (17.65)	2 (33.33)		
Yes	14 (82.35)	4 (66.67)		

*Means a statistically significant difference.

Based on these findings, to assess their independent effects, variables showing statistical significance in the univariate analysis (AMS and WBC count) were entered into a binary logistic regression model (Table 4). The multivariate analysis retained AMS as an independent risk factor for mortality (OR = 14.03, 95% CI: 1.19–165.08, *P* = 0.036). In contrast, the WBC count was not an independent predictor (OR = 1.09, 95% CI: 0.81–1.48, *P* = 0.574). Thus, AMS was identified as an independent predictor of patient prognosis.

**TABLE 4:** Multivariate analysis of factors associated with mortality.

Variable	β	SE	X^2^	OR	95% CI	*P*
White blood cell	0.09	0.16	0.32	1.09	0.81–1.48	0.574
AMS	−2.64	1.26	4.41	14.03	1.19–165.08	0.036*

*Means a statistically significant difference.

## 4 Discussion

This retrospective analysis of 23 CP-MIH patients revealed a high mortality rate (73.9%) and a median survival of merely 13.5 h in non-survivors, with AMS emerging as a strong independent predictor of fatal outcome. Bunderen et al. reported a mortality rate of 80% among 40 patients with CP-MIH, with a median time from onset to death of only 8 h (27). In our cohort, the mortality rate among hematologic patients is slightly lower and the median survival time was longer than those reported by Bunderen et al. This discrepancy may be attributed to the fact that all cases in the Bunderen cohort occurred prior to 2010, supporting the influence of era-related factors on outcomes. Consistent with this, all 10 hematologic patients diagnosed before 2010 in our cohort died, whereas survivors emerged among cases reported after 2014. Improvements in diagnostic speed (e.g., rapid PCR), earlier intensive care support, and more aggressive antimicrobial combinations in recent years may have contributed to a modest survival improvement (3, 28, 29).

The mean age of patients in this cohort was 45.87 ± 17.94 years, which was significantly lower than that reported in previous studies of general CP-MIH populations (61–66.5 years) (3, 27, 30). The proportion of male patients (60.8%) was consistent with previously reported rates (58.1%–60.0%) (3). The gastrointestinal tract was the primary route of infection (47.8%), aligning with existing literature which identifies it as the most common site of origin for CP-MIH (27). This supports the notion that gastrointestinal infections may pose a higher risk of triggering hemolysis. Clinically, CP-MIH patients often present with AMS, shock, gas gangrene, and hematuria (3). However, only four cases in this cohort were confirmed to have gas gangrene. Therefore, it was excluded from prognostic analysis. Suzaki et al. noted the prevalence of AMS in their cohort, its prognostic significance remained unquantified (3). Our study not only reaffirm the grave prognosis associated with CP-MIH but, more importantly, identifies AMS as a quantifiable early-warning sign that is readily assessable at the bedside. The mortality rate among patients with AMS was as high as 92.8% (13/14), underscoring its critical value as a prognostic indicator. While an elevated WBC count was associated with death in univariate analysis, it was not an independent predictor in the multivariate model. This apparent discrepancy may stem from the small sample size or the unique pathophysiology of CP infection. The higher WBC count observed in non-survivors likely reflects a stress response to severe infection; however, its absolute value remained profoundly low (<1.0 × 10^9^/L), indicating persistent immunosuppression. Thus, the WBC count may be a marker of overall disease severity, whereas AMS more directly signals the imminent fatal impact of CP-MIH.

**FIGURE 1:**
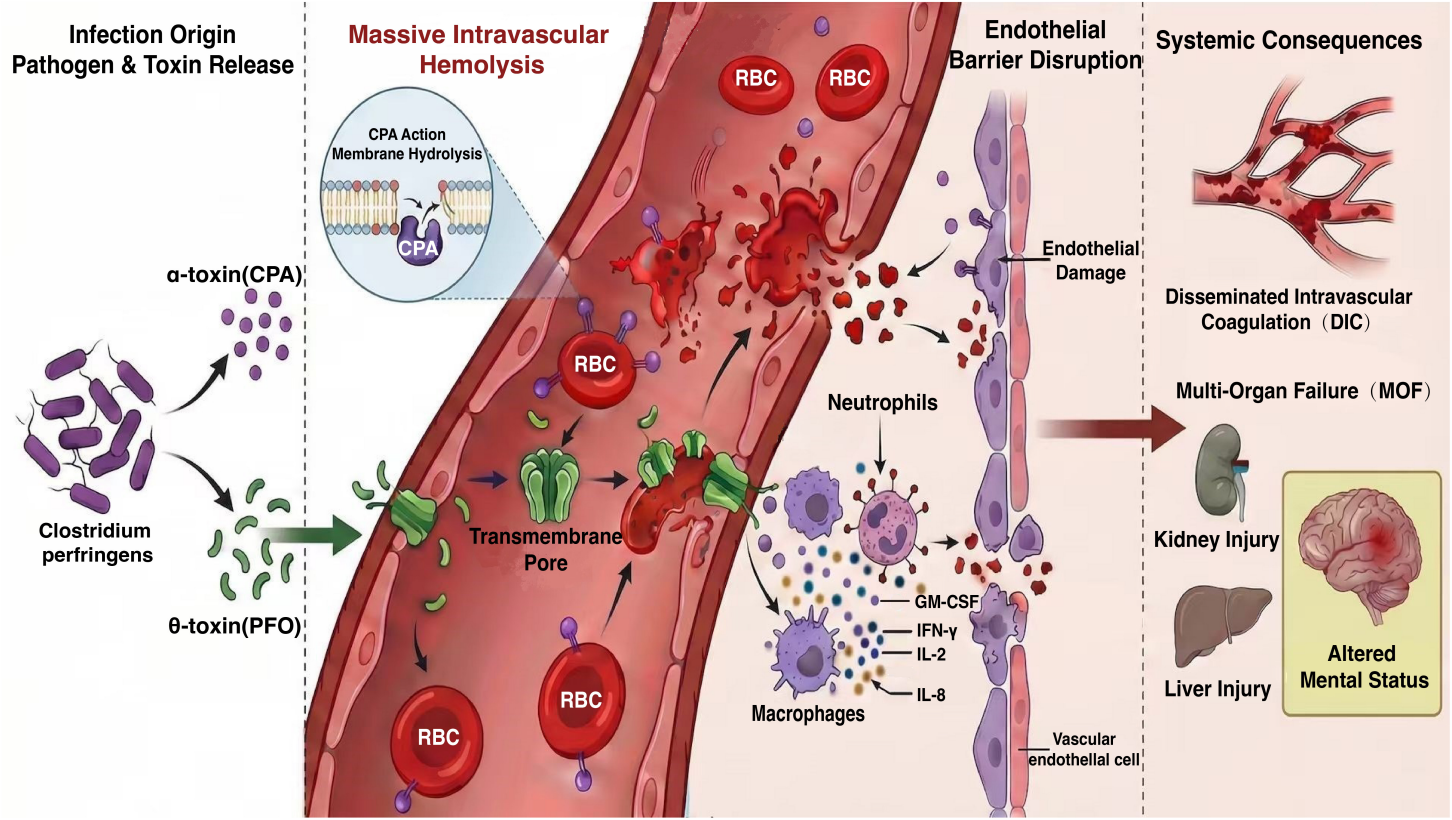
Proposed pathogenesis of *Clostridium perfringens*-induced massive intravascular hemolysis. This schematic illustrates the postulated “double-hit” pathogenic cascade of CP-MIH. (1) Infection and toxin release: rapid proliferation of *C. perfringens* and release of major exotoxins (CPA, PFO). (2) Fulminant hemolysis and direct damage: CPA hydrolyzes RBC membranes, while PFO forms pores, synergistically causing massive intravascular hemolysis and direct cellular injury. (3) Vicious cycle and systemic storm: hemolysis products (DAMPs) and toxins activate immune cells (e.g., neutrophils, macrophages), triggering a cytokine storm and endothelial damage. This cascade establishes a vicious cycle that exacerbates hemolysis and injury. (4) End-stage organ failure: the convergent effects of endothelial dysfunction, coagulopathy (DIC), and systemic inflammation lead to multi-organ failure. CPA, α-toxin; PFO, θ-toxin; RBC, red blood cell; DIC, disseminated intravascular coagulation; MOF, multi-organ failure; DAMP, damage-associated molecular pattern.

The pathogenesis of CP-MIH remains incompletely understood. Current evidence suggests it is primarily associated with α-toxin (CPA) and θ-toxin (PFO). CPA lyses red blood cells (RBCs) and other host cells by hydrolyzing membrane phosphatidylcholine and phospholipids, while simultaneously activating immune cells to release pro-inflammatory cytokines such as interferon-γ, interleukin (IL)-2, IL-8, and granulocyte-macrophage colony-stimulating factor (GM-CSF), thereby initiating a cytokine storm (31). Meanwhile, PFO exerts its effect primarily by forming transmembrane pores, leading to direct cell lysis (32). *In vitro* studies suggest that PFO possesses stronger hemolytic and pro-inflammatory activity than CPA (31), indicating it may play a dominant role in triggering MIH. Furthermore, toxin-mediated endothelial injury may exacerbate hemolysis, creating a self-perpetuating vicious cycle. This pathogenic cascade can be conceptualized as a “double-hit” model (Figure 1). The first hit involves rapid bacterial proliferation and the massive release of toxins, directly causing fulminant intravascular hemolysis. The second hit ensues as hemolysis products (e.g., free hemoglobin) and toxins act synergistically to induce a systemic inflammatory response (cytokine storm) and disseminated intravascular coagulation, culminating in irreversible multi-organ failure. This rapid sequence results in an exceedingly narrow therapeutic window. In the present study, over 76% of deceased patients did not receive a pathogen-confirmed diagnosis before death, underscoring the profound diagnostic and therapeutic challenges posed by CP-MIH in clinical practice.

*Clostridium perfringens* exhibits extremely rapid proliferation, with a doubling time of approximately 7 minutes (33). In contrast, conventional blood cultures require an average of 16.9 h to yield positive results (34), a timeframe that significantly exceeds the median survival time of patients with CP-MIH (27). Therefore, neutropenic patients with fever, gastrointestinal symptoms, and sudden unexplained hemolysis should be highly suspected of having a CP infection. Studies have demonstrated that real-time quantitative PCR targeting the CPA gene can detect the pathogen within 3 h (29), offering markedly improved sensitivity and timeliness compared to traditional culture methods (35, 36). Imaging studies that reveal findings such as gas gangrene or hepatic gas abscesses may also provide valuable clues for early diagnosis.

Currently, there is no specific therapy for CP-MIH. Prompt empiric combination antibiotic therapy is paramount. CP might remain sensitive to antibiotics such as penicillin and carbapenems, but these agents cannot neutralize toxins already released by CP (30). Clindamycin and metronidazole can rapidly inhibit the activity of α-toxin (CPA) (37, 38), and evidence suggests that clindamycin combined with penicillin may improve survival outcomes (3). Based on the available evidence and the rapid progression of CP-MIH, we propose a stepwise clinical approach: (i). high clinical suspicion in neutropenic patients with acute hemolysis; (ii). immediate collection of blood cultures and rapid molecular testing if available; (iii). prompt initiation of combination antibiotic therapy (e.g., high-dose penicillin G or carbapenem plus clindamycin); (iv). early admission to the intensive care unit for hemodynamic and organic support; (v). consideration of adjunctive measures such as blood purification/continuous renal replacement therapy (CRRT) to remove circulating toxins, and surgical debridement if a focal infection is identified (39). Hyperbaric oxygen therapy may also help mitigate organ damage in selected cases (40).

Emerging strategies directly targeting the pathogenic toxins offer new hope for treating CP-MIH in recent years. A key finding is that CPA-induced hemolysis depends on purinergic (P2) receptor activation and can be potently inhibited by P2 receptor antagonists such as PPADS (41). Beyond receptor blockade, anti-PFO antibodies, particularly when combined with IL-6 receptor antagonists, offer a strategy to simultaneously curb hemolysis and mitigate the cytokine storm (42). Furthermore, natural compounds such as flavonoids (e.g., amentoflavone) and proton pump inhibitors (e.g., rabeprazole) have demonstrated direct anti-toxin effects in preclinical studies (43, 44). Another innovative approach involves antimicrobial peptide-based nanocarriers designed specifically against CP, which have shown efficacy in animal studies (45). These diverse strategies, though preclinical, highlight critical pathways for intervention, mandating future research to bridge the gap to clinical application.

This study has several limitations that should be considered when interpreting the results. First, the retrospective design and reliance on case reports and small case series from the literature may introduce selection bias and limit the generalizability of the findings. Second, the small sample size, though understandable given the rarity of CP-MIH, reduces the statistical power of the analysis, potentially obscuring other significant prognostic factors. Third, heterogeneity in clinical documentation across different institutions and time periods may have led to inconsistencies in data collection, especially regarding laboratory values, timing of interventions, and cause of death. Finally, the evolution of diagnostic techniques and supportive care over the decades represents a confounding factor that could influence survival trends and complicate direct comparisons.

## 5 Conclusion and future directions

*Clostridium perfringens*- massive intravascular hemolysis is a highly fatal complication in patients with hematologic malignancies, marked by abrupt onset and exceedingly high mortality. AMS serves as an independent predictor of poor prognosis. Clinicians should be highly suspicious of CP infection in neutropenic patients presenting with hemolysis, initiate prompt combination antibiotic therapy covering CP (e.g., penicillin plus clindamycin), and facilitate early admission to the intensive care unit for advanced support. To build upon these essential clinical steps and further improve survival, future research must prioritize the following directions: (I). Diagnostic innovation: development and widespread validation of point-of-care rapid diagnostic tests are essential to shorten the time to pathogen identification. (II). Therapeutic novelty: beyond antibiotics, future efforts must focus on toxin-targeting strategies to directly counteract the pathogenic cascade. Promising preclinical avenues include P2 receptor antagonists, neutralizing antibodies and the exploration of repurposed agents with anti-toxin properties. (III). Clinical translation: well-designed multicenter prospective studies, though challenging given the rarity of CP-MIH, are needed to formally evaluate the efficacy of combination therapies, adjunctive interventions and the potential of novel anti-toxin agents in improving survival. (IV). Prevention and risk stratification: further investigation into the gut microbiota dynamics and host factors in hematologic patients may identify modifiable risks for CP colonization and subsequent invasive infection.

## Data Availability

The original contributions presented in this study are included in this article/supplementary material, further inquiries can be directed to the corresponding author.
